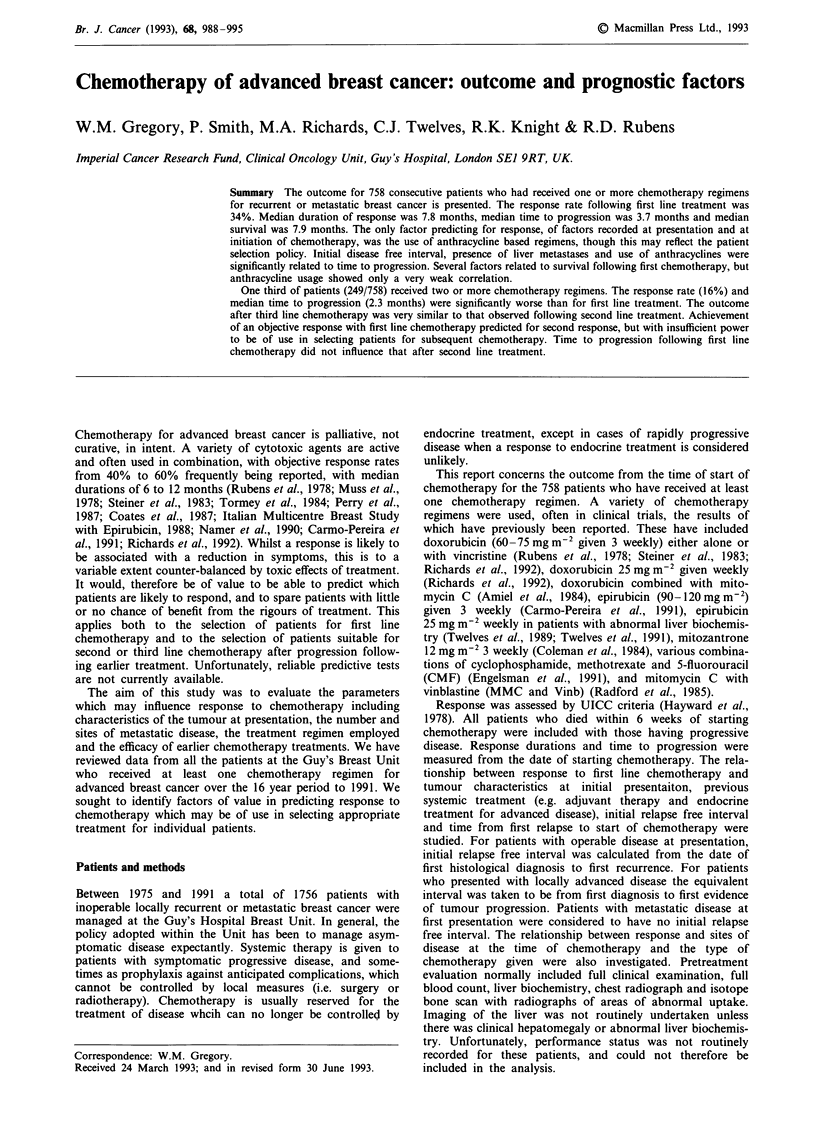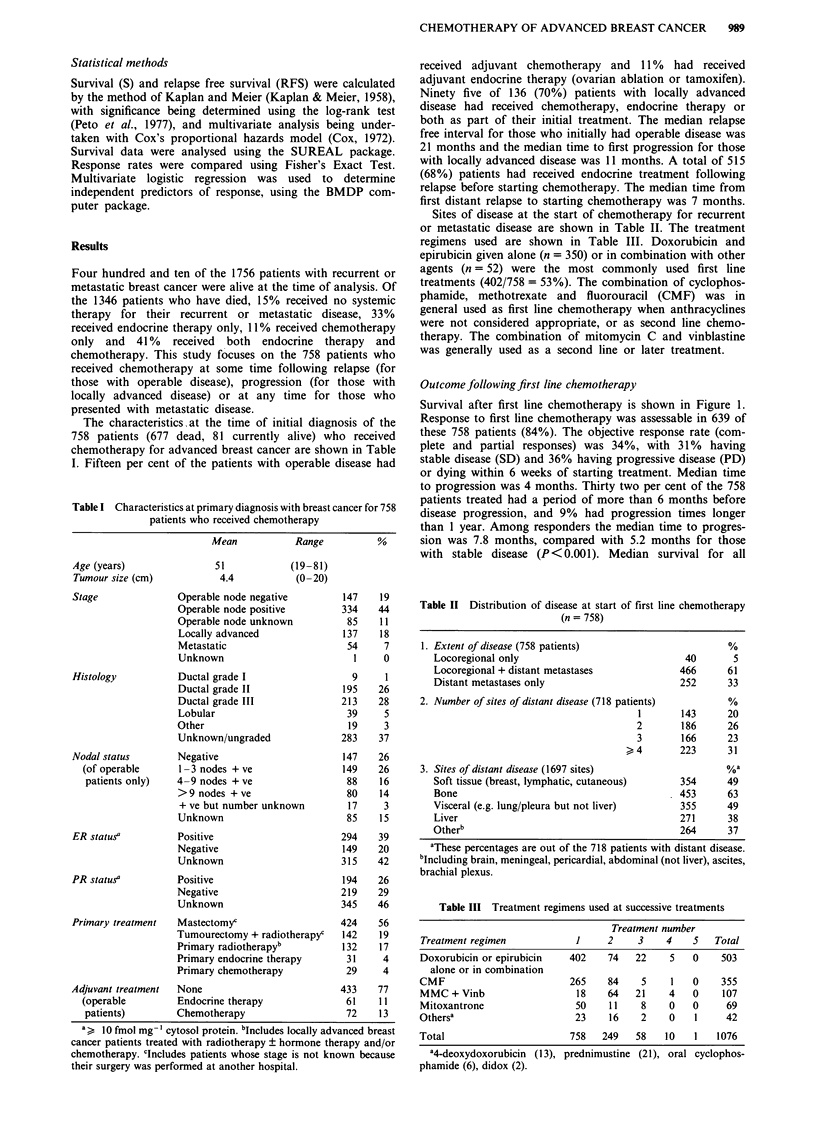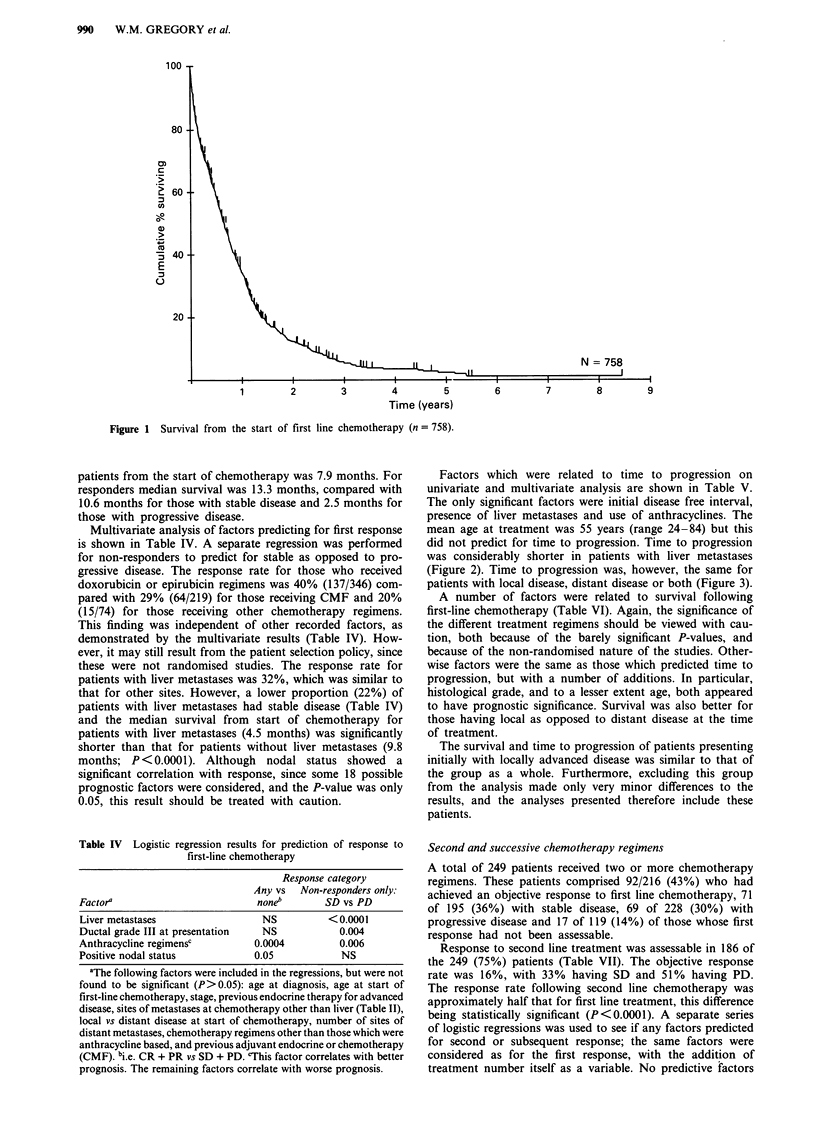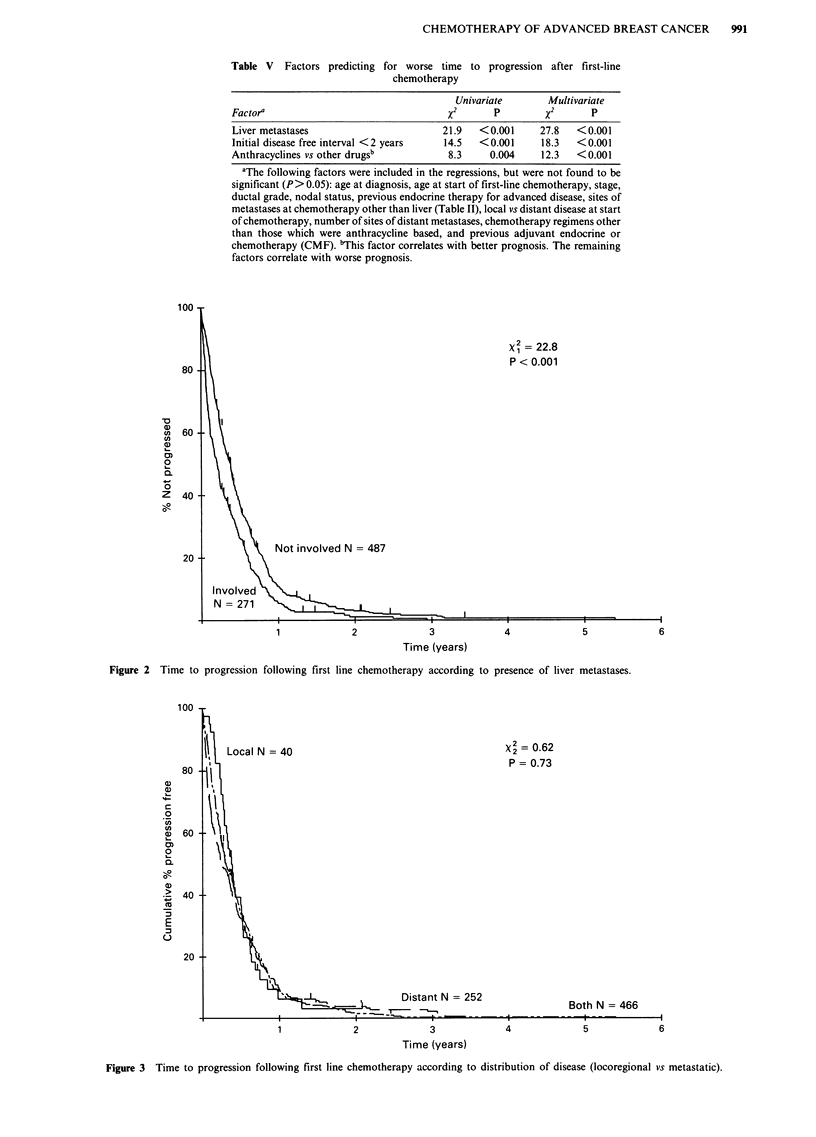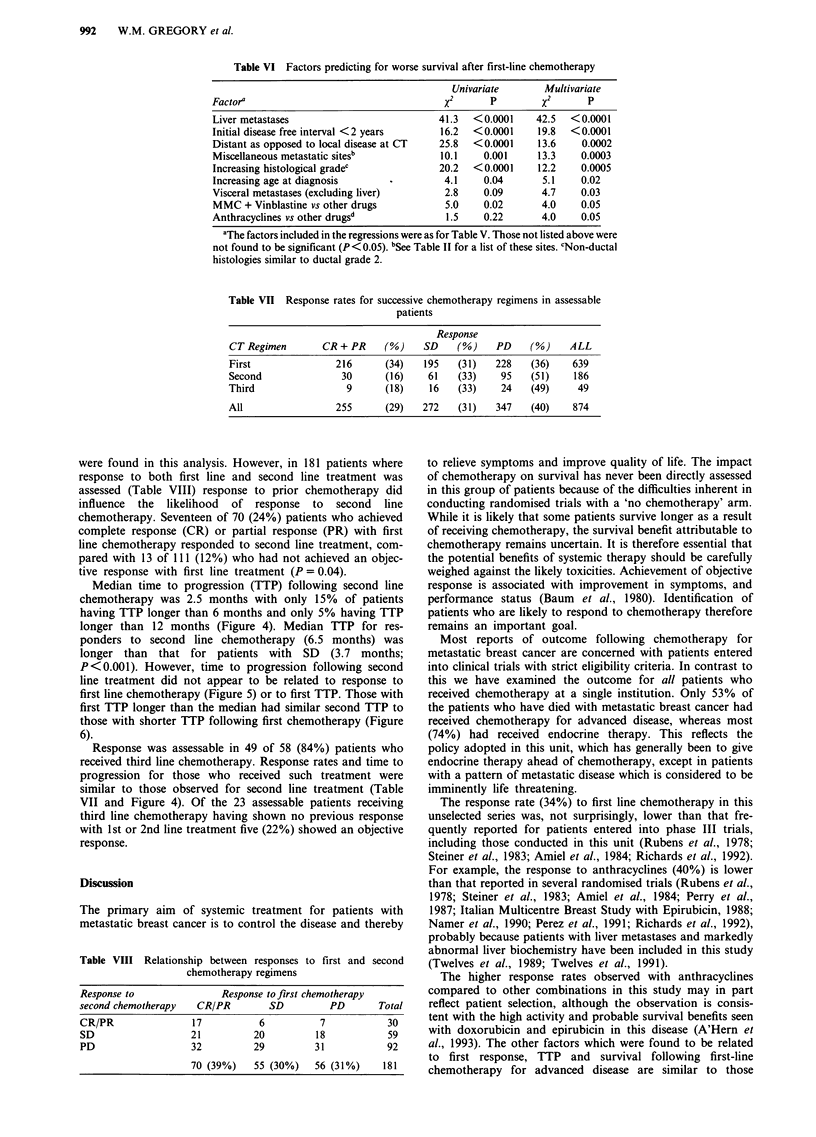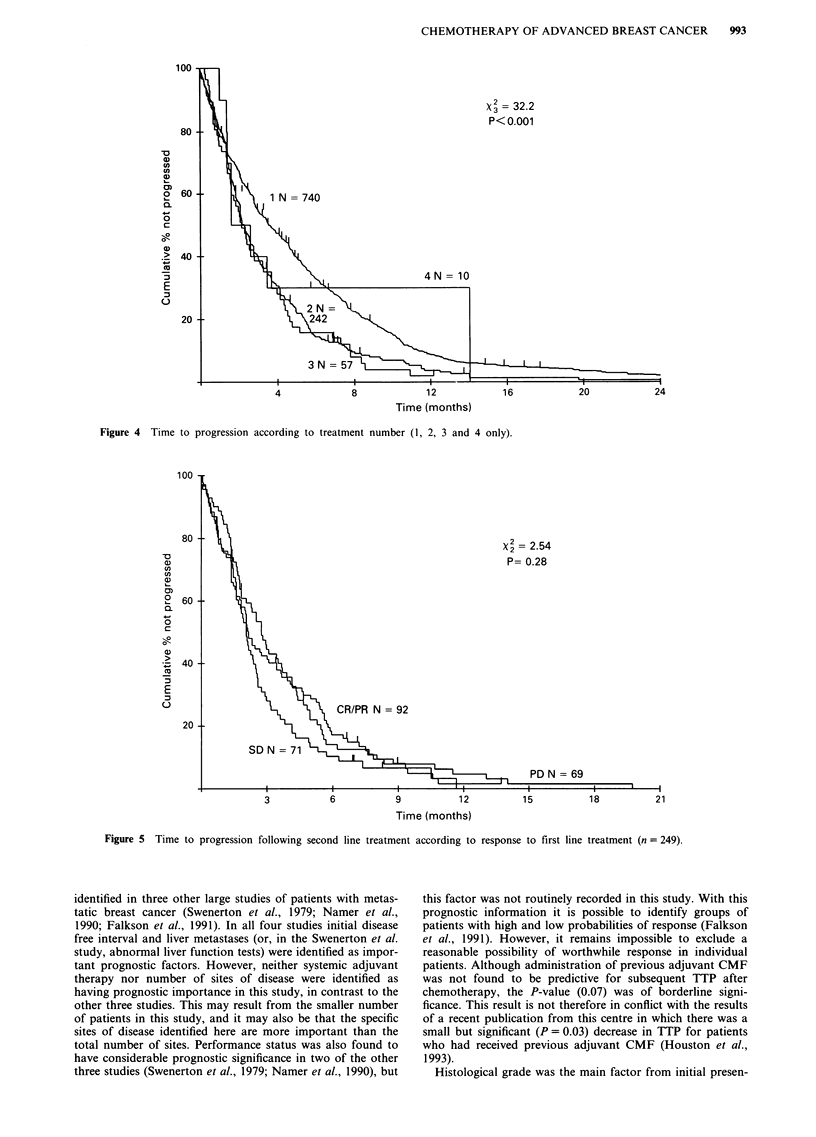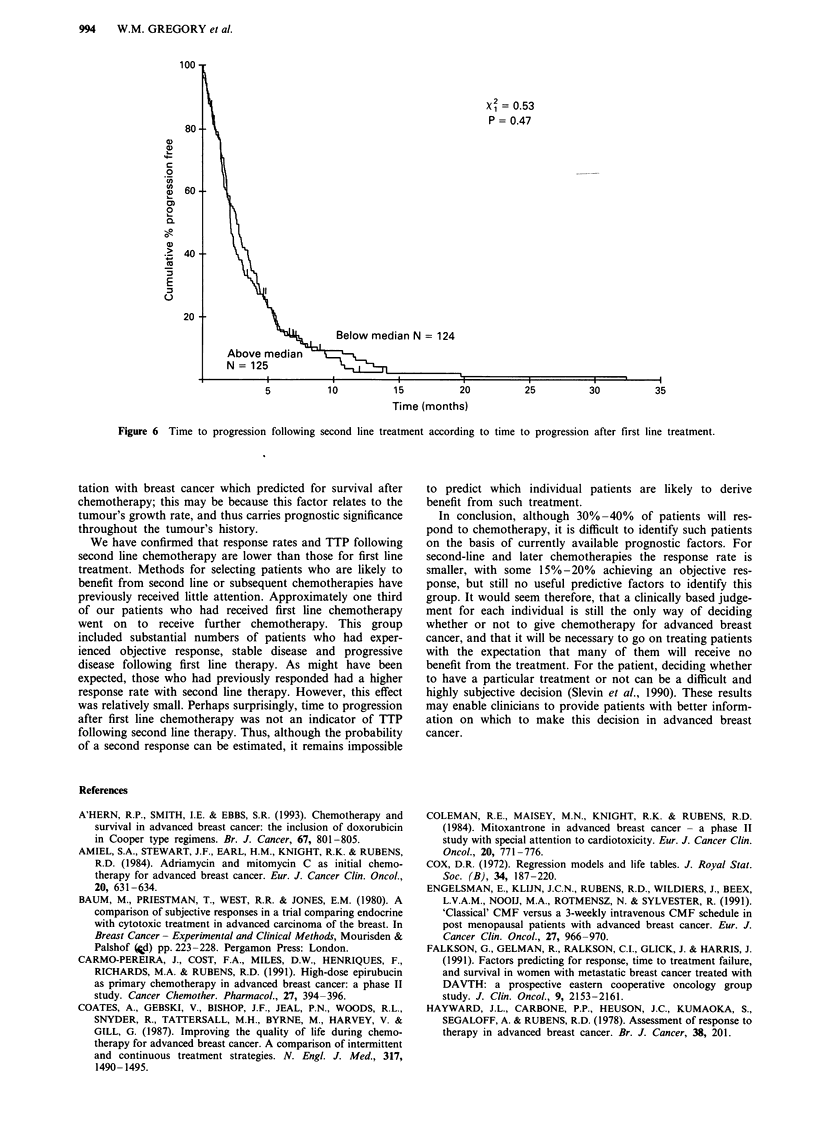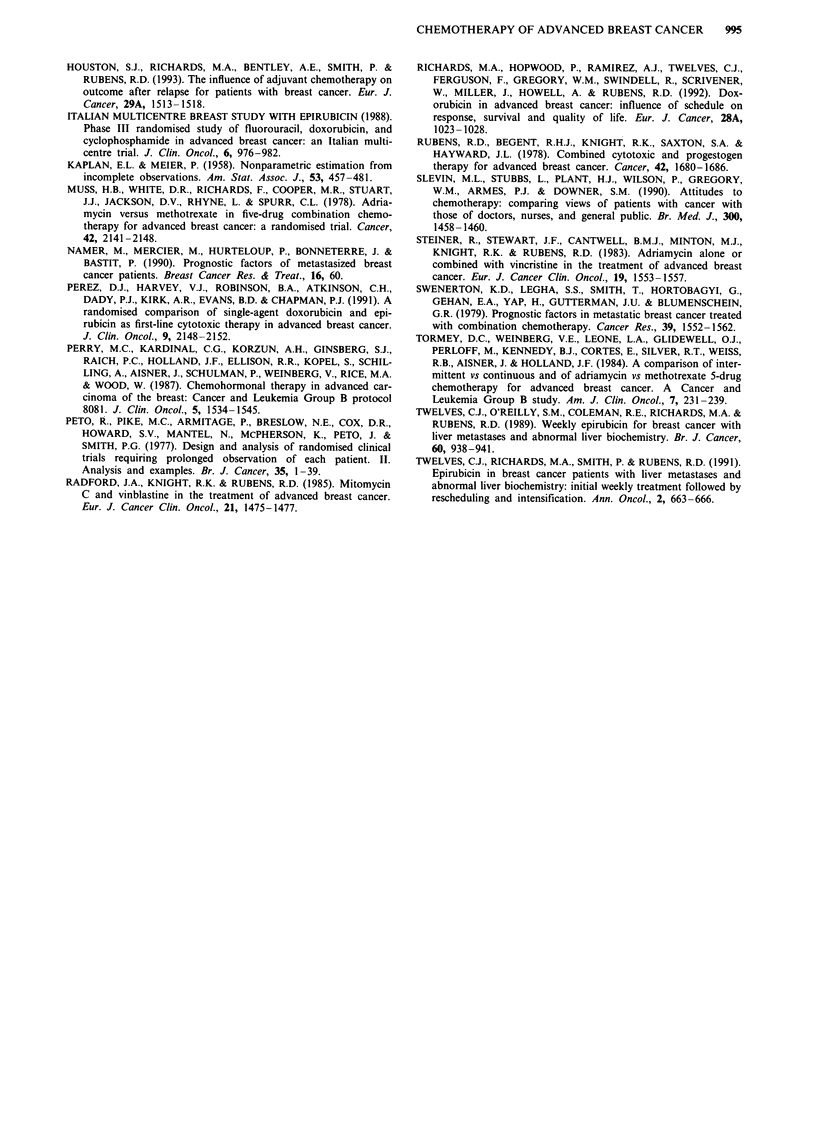# Chemotherapy of advanced breast cancer: outcome and prognostic factors.

**DOI:** 10.1038/bjc.1993.467

**Published:** 1993-11

**Authors:** W. M. Gregory, P. Smith, M. A. Richards, C. J. Twelves, R. K. Knight, R. D. Rubens

**Affiliations:** Imperial Cancer Research Fund, Guy's Hospital, London, UK.

## Abstract

The outcome for 758 consecutive patients who had received one or more chemotherapy regimens for recurrent or metastatic breast cancer is presented. The response rate following first line treatment was 34%. Median duration of response was 7.8 months, median time to progression was 3.7 months and median survival was 7.9 months. The only factor predicting for response, of factors recorded at presentation and at initiation of chemotherapy, was the use of anthracycline based regimens, though this may reflect the patient selection policy. Initial disease free interval, presence of liver metastases and use of anthracyclines were significantly related to time to progression. Several factors related to survival following first chemotherapy, but anthracycline usage showed only a very weak correlation. One third of patients (249/758) received two or more chemotherapy regimens. The response rate (16%) and median time to progression (2.3 months) were significantly worse than for first line treatment. The outcome after third line chemotherapy was very similar to that observed following second line treatment. Achievement of an objective response with first line chemotherapy predicted for second response, but with insufficient power to be of use in selecting patients for subsequent chemotherapy. Time to progression following first line chemotherapy did not influence that after second line treatment.


Br. J. Cancer (1993), 68, 988–995 © Macmillan Press Ltd., 1993

### Chemotherapy of advanced breast cancer: outcome and prognostic factors

W.M. Gregory, P. Smith, M.A. Richards, C.J. Twelves, R.K. Knight & R.D. Rubens

*Imperial Cancer Research Fund, Clinical Oncology Unit, Guy's Hospital, London SE1 9RT, UK.*

**Summary** The outcome for 758 consecutive patients who had received one or more chemotherapy regimens for recurrent or metastatic breast cancer is presented. The response rate following first line treatment was 34%. Median duration of response was 7.8 months, median time to progression was 3.7 months and median survival was 7.9 months. The only factor predicting for response, of factors recorded at presentation and at initiation of chemotherapy, was the use of anthracycline based regimens, though this may reflect the patient selection policy. Initial disease free interval, presence of liver metastases and use of anthracyclines were significantly related to time to progression. Several factors related to survival following first chemotherapy, but anthracycline usage showed only a very weak correlation.

One third of patients (249/758) received two or more chemotherapy regimens. The response rate (16%) and median time to progression (2.3 months) were significantly worse than for first line treatment. The outcome after third line chemotherapy was very similar to that observed following second line treatment. Achievement of an objective response with first line chemotherapy predicted for second response, but with insufficient power to be of use in selecting patients for subsequent chemotherapy. Time to progression following first line chemotherapy did not influence that after second line treatment.

Chemotherapy for advanced breast cancer is palliative, not curative, in intent. A variety of cytotoxic agents are active and often used in combination, with objective response rates from 40% to 60% frequently being reported, with median durations of 6 to 12 months (Rubens *et al.*, 1978; Muss *et al.*, 1978; Steiner *et al.*, 1983; Tormey *et al.*, 1984; Perry *et al.*, 1987; Coates *et al.*, 1987; Italian Multicentre Breast Study with Epirubicin, 1988; Namer *et al.*, 1990; Carmo-Pereira *et al.*, 1991; Richards *et al.*, 1992). Whilst a response is likely to be associated with a reduction in symptoms, this is to a variable extent counter-balanced by toxic effects of treatment. It would, therefore be of value to be able to predict which patients are likely to respond, and to spare patients with little or no chance of benefit from the rigours of treatment. This applies both to the selection of patients for first line chemotherapy and to the selection of patients suitable for second or third line chemotherapy after progression following earlier treatment. Unfortunately, reliable predictive tests are not currently available.

The aim of this study was to evaluate the parameters which may influence response to chemotherapy including characteristics of the tumour at presentation, the number and sites of metastatic disease, the treatment regimen employed and the efficacy of earlier chemotherapy treatments. We have reviewed data from all the patients at the Guy's Breast Unit who received at least one chemotherapy regimen for advanced breast cancer over the 16 year period to 1991. We sought to identify factors of value in predicting response to chemotherapy which may be of use in selecting appropriate treatment for individual patients.

### Patients and methods

Between 1975 and 1991 a total of 1756 patients with inoperable locally recurrent or metastatic breast cancer were managed at the Guy's Hospital Breast Unit. In general, the policy adopted within the Unit has been to manage asymptomatic disease expectantly. Systemic therapy is given to patients with symptomatic progressive disease, and sometimes as prophylaxis against anticipated complications, which cannot be controlled by local measures (i.e. surgery or radiotherapy). Chemotherapy is usually reserved for the treatment of disease whcih can no longer be controlled by endocrine treatment, except in cases of rapidly progressive disease when a response to endocrine treatment is considered unlikely.

This report concerns the outcome from the time of start of chemotherapy for the 758 patients who have received at least one chemotherapy regimen. A variety of chemotherapy regimens were used, often in clinical trials, the results of which have previously been reported. These have included doxorubicin (60–75 mg $m^{-2}$ given 3 weekly) either alone or with vincristine (Rubens *et al.*, 1978; Steiner *et al.*, 1983; Richards *et al.*, 1992), doxorubicin 25 mg $m^{-2}$ given weekly (Richards *et al.*, 1992), doxorubicin combined with mitomycin C (Amiel *et al.*, 1984), epirubicin (90–120 mg $m^{-2}$) given 3 weekly (Carmo-Pereira *et al.*, 1991), epirubicin 25 mg $m^{-2}$ weekly in patients with abnormal liver biochemistry (Twelves *et al.*, 1989; Twelves *et al.*, 1991), mitozantrone 12 mg $m^{-2}$ 3 weekly (Coleman *et al.*, 1984), various combinations of cyclophosphamide, methotrexate and 5-fluorouracil (CMF) (Engelsman *et al.*, 1991), and mitomycin C with vinblastine (MMC and Vinb) (Radford *et al.*, 1985).

Response was assessed by UICC criteria (Hayward *et al.*, 1978). All patients who died within 6 weeks of starting chemotherapy were included with those having progressive disease. Response durations and time to progression were measured from the date of starting chemotherapy. The relationship between response to first line chemotherapy and tumour characteristics at initial presentaiton, previous systemic treatment (e.g. adjuvant therapy and endocrine treatment for advanced disease), initial relapse free interval and time from first relapse to start of chemotherapy were studied. For patients with operable disease at presentation, initial relapse free interval was calculated from the date of first histological diagnosis to first recurrence. For patients who presented with locally advanced disease the equivalent interval was taken to be from first diagnosis to first evidence of tumour progression. Patients with metastatic disease at first presentation were considered to have no initial relapse free interval. The relationship between response and sites of disease at the time of chemotherapy and the type of chemotherapy given were also investigated. Pretreatment evaluation normally included full clinical examination, full blood count, liver biochemistry, chest radiograph and isotope bone scan with radiographs of areas of abnormal uptake. Imaging of the liver was not routinely undertaken unless there was clinical hepatomegaly or abnormal liver biochemistry. Unfortunately, performance status was not routinely recorded for these patients, and could not therefore be included in the analysis.

Correspondence: W.M. Gregory.
Received 24 March 1993; and in revised form 30 June 1993.

*Statistical methods*

Survival (S) and relapse free survival (RFS) were calculated by the method of Kaplan and Meier (Kaplan & Meier, 1958), with significance being determined using the log-rank test (Peto *et al.*, 1977), and multivariate analysis being undertaken with Cox's proportional hazards model (Cox, 1972). Survival data were analysed using the SUREAL package. Response rates were compared using Fisher's Exact Test. Multivariate logistic regression was used to determine independent predictors of response, using the BMDP computer package.

### Results

Four hundred and ten of the 1756 patients with recurrent or metastatic breast cancer were alive at the time of analysis. Of the 1346 patients who have died, 15% received no systemic therapy for their recurrent or metastatic disease, 33% received endocrine therapy only, 11% received chemotherapy only and 41% received both endocrine therapy and chemotherapy. This study focuses on the 758 patients who received chemotherapy at some time following relapse (for those with operable disease), progression (for those with locally advanced disease) or at any time for those who presented with metastatic disease.

The characteristics at the time of initial diagnosis of the 758 patients (677 dead, 81 currently alive) who received chemotherapy for advanced breast cancer are shown in Table I. Fifteen per cent of the patients with operable disease had received adjuvant chemotherapy and 11% had received adjuvant endocrine therapy (ovarian ablation or tamoxifen). Ninety five of 136 (70%) patients with locally advanced disease had received chemotherapy, endocrine therapy or both as part of their initial treatment. The median relapse free interval for those who initially had operable disease was 21 months and the median time to first progression for those with locally advanced disease was 11 months. A total of 515 (68%) patients had received endocrine treatment following relapse before starting chemotherapy. The median time from first distant relapse to starting chemotherapy was 7 months.

Sites of disease at the start of chemotherapy for recurrent or metastatic disease are shown in Table II. The treatment regimens used are shown in Table III. Doxorubicin and epirubicin given alone ($n = 350$) or in combination with other agents ($n = 52$) were the most commonly used first line treatments (402/758 = 53%). The combination of cyclophosphamide, methotrexate and fluorouracil (CMF) was in general used as first line chemotherapy when anthracyclines were not considered appropriate, or as second line chemotherapy. The combination of mitomycin C and vinblastine was generally used as a second line or later treatment.

**Table I** Characteristics at primary diagnosis with breast cancer for 758 patients who received chemotherapy

| | | *Mean* | *Range* | % |
|---|---|---|---|---|
| *Age* (years) | | 51 | (19–81) | |
| *Tumour size* (cm) | | 4.4 | (0–20) | |
| *Stage* | Operable node negative | | 147 | 19 |
| | Operable node positive | | 334 | 44 |
| | Operable node unknown | | 85 | 11 |
| | Locally advanced | | 137 | 18 |
| | Metastatic | | 54 | 7 |
| | Unknown | | 1 | 0 |
| *Histology* | Ductal grade I | | 9 | 1 |
| | Ductal grade II | | 195 | 26 |
| | Ductal grade III | | 213 | 28 |
| | Lobular | | 39 | 5 |
| | Other | | 19 | 3 |
| | Unknown/ungraded | | 283 | 37 |
| *Nodal status* (of operable patients only) | Negative | | 147 | 26 |
| | 1–3 nodes + ve | | 149 | 26 |
| | 4–9 nodes + ve | | 88 | 16 |
| | >9 nodes + ve | | 80 | 14 |
| | + ve but number unknown | | 17 | 3 |
| | Unknown | | 85 | 15 |
| *ER status*[a] | Positive | | 294 | 39 |
| | Negative | | 149 | 20 |
| | Unknown | | 315 | 42 |
| *PR status*[a] | Positive | | 194 | 26 |
| | Negative | | 219 | 29 |
| | Unknown | | 345 | 46 |
| *Primary treatment* | Mastectomy[c] | | 424 | 56 |
| | Tumourectomy + radiotherapy[c] | | 142 | 19 |
| | Primary radiotherapy[b] | | 132 | 17 |
| | Primary endocrine therapy | | 31 | 4 |
| | Primary chemotherapy | | 29 | 4 |
| *Adjuvant treatment* (operable patients) | None | | 433 | 77 |
| | Endocrine therapy | | 61 | 11 |
| | Chemotherapy | | 72 | 13 |

[a]$\geqslant$ 10 fmol $mg^{-1}$ cytosol protein. [b]Includes locally advanced breast cancer patients treated with radiotherapy ± hormone therapy and/or chemotherapy. [c]Includes patients whose stage is not known because their surgery was performed at another hospital.

*Outcome following first line chemotherapy*

Survival after first line chemotherapy is shown in Figure 1. Response to first line chemotherapy was assessable in 639 of these 758 patients (84%). The objective response rate (complete and partial responses) was 34%, with 31% having stable disease (SD) and 36% having progressive disease (PD) or dying within 6 weeks of starting treatment. Median time to progression was 4 months. Thirty two per cent of the 758 patients treated had a period of more than 6 months before disease progression, and 9% had progression times longer than 1 year. Among responders the median time to progression was 7.8 months, compared with 5.2 months for those with stable disease ($P < 0.001$). Median survival for all

**Table II** Distribution of disease at start of first line chemotherapy ($n = 758$)

| | | |
|---|---|---|
| 1. *Extent of disease* (758 patients) | | % |
| Locoregional only | 40 | 5 |
| Locoregional + distant metastases | 466 | 61 |
| Distant metastases only | 252 | 33 |
| 2. *Number of sites of distant disease* (718 patients) | | % |
| 1 | 143 | 20 |
| 2 | 186 | 26 |
| 3 | 166 | 23 |
| $\geqslant 4$ | 223 | 31 |
| 3. *Sites of distant disease* (1697 sites) | | %[a] |
| Soft tissue (breast, lymphatic, cutaneous) | 354 | 49 |
| Bone | 453 | 63 |
| Visceral (e.g. lung/pleura but not liver) | 355 | 49 |
| Liver | 271 | 38 |
| Other[b] | 264 | 37 |

[a]These percentages are out of the 718 patients with distant disease. [b]Including brain, meningeal, pericardial, abdominal (not liver), ascites, brachial plexus.

**Table III** Treatment regimens used at successive treatments

| | Treatment number | | | | | |
|---|---|---|---|---|---|---|
| *Treatment regimen* | *1* | *2* | *3* | *4* | *5* | *Total* |
| Doxorubicin or epirubicin alone or in combination | 402 | 74 | 22 | 5 | 0 | 503 |
| CMF | 265 | 84 | 5 | 1 | 0 | 355 |
| MMC + Vinb | 18 | 64 | 21 | 4 | 0 | 107 |
| Mitoxantrone | 50 | 11 | 8 | 0 | 0 | 69 |
| Others[a] | 23 | 16 | 2 | 0 | 1 | 42 |
| Total | 758 | 249 | 58 | 10 | 1 | 1076 |

[a]4-deoxydoxorubicin (13), prednimustine (21), oral cyclophosphamide (6), didox (2).

**Figure 1** Survival from the start of first line chemotherapy ($n = 758$).

patients from the start of chemotherapy was 7.9 months. For responders median survival was 13.3 months, compared with 10.6 months for those with stable disease and 2.5 months for those with progressive disease.

Multivariate analysis of factors predicting for first response is shown in Table IV. A separate regression was performed for non-responders to predict for stable as opposed to progressive disease. The response rate for those who received doxorubicin or epirubicin regimens was 40% (137/346) compared with 29% (64/219) for those receiving CMF and 20% (15/74) for those receiving other chemotherapy regimens. This finding was independent of other recorded factors, as demonstrated by the multivariate results (Table IV). However, it may still result from the patient selection policy, since these were not randomised studies. The response rate for patients with liver metastases was 32%, which was similar to that for other sites. However, a lower proportion (22%) of patients with liver metastases had stable disease (Table IV) and the median survival from start of chemotherapy for patients with liver metastases (4.5 months) was significantly shorter than that for patients without liver metastases (9.8 months; $P < 0.0001$). Although nodal status showed a significant correlation with response, since some 18 possible prognostic factors were considered, and the $P$-value was only 0.05, this result should be treated with caution.

**Table IV** Logistic regression results for prediction of response to first-line chemotherapy

| | Response category | |
|---|---|---|
| *Factor*[a] | *Any* vs *none*[b] | *Non-responders only:* *SD* vs *PD* |
| Liver metastases | NS | <0.0001 |
| Ductal grade III at presentation | NS | 0.004 |
| Anthracycline regimens[c] | 0.0004 | 0.006 |
| Positive nodal status | 0.05 | NS |

[a]The following factors were included in the regressions, but were not found to be significant ($P > 0.05$): age at diagnosis, age at start of first-line chemotherapy, stage, previous endocrine therapy for advanced disease, sites of metastases at chemotherapy other than liver (Table II), local *vs* distant disease at start of chemotherapy, number of sites of distant metastases, chemotherapy regimens other than those which were anthracycline based, and previous adjuvant endocrine or chemotherapy (CMF). [b]i.e. CR + PR *vs* SD + PD. [c]This factor correlates with better prognosis. The remaining factors correlate with worse prognosis.

Factors which were related to time to progression on univariate and multivariate analysis are shown in Table V. The only significant factors were initial disease free interval, presence of liver metastases and use of anthracyclines. The mean age at treatment was 55 years (range 24–84) but this did not predict for time to progression. Time to progression was considerably shorter in patients with liver metastases (Figure 2). Time to progression was, however, the same for patients with local disease, distant disease or both (Figure 3).

A number of factors were related to survival following first-line chemotherapy (Table VI). Again, the significance of the different treatment regimens should be viewed with caution, both because of the barely significant $P$-values, and because of the non-randomised nature of the studies. Otherwise factors were the same as those which predicted time to progression, but with a number of additions. In particular, histological grade, and to a lesser extent age, both appeared to have prognostic significance. Survival was also better for those having local as opposed to distant disease at the time of treatment.

The survival and time to progression of patients presenting initially with locally advanced disease was similar to that of the group as a whole. Furthermore, excluding this group from the analysis made only very minor differences to the results, and the analyses presented therefore include these patients.

*Second and successive chemotherapy regimens*

A total of 249 patients received two or more chemotherapy regimens. These patients comprised 92/216 (43%) who had achieved an objective response to first line chemotherapy, 71 of 195 (36%) with stable disease, 69 of 228 (30%) with progressive disease and 17 of 119 (14%) of those whose first response had not been assessable.

Response to second line treatment was assessable in 186 of the 249 (75%) patients (Table VII). The objective response rate was 16%, with 33% having SD and 51% having PD. The response rate following second line chemotherapy was approximately half that for first line treatment, this difference being statistically significant ($P < 0.0001$). A separate series of logistic regressions was used to see if any factors predicted for second or subsequent response; the same factors were considered as for the first response, with the addition of treatment number itself as a variable. No predictive factors

**Table V** Factors predicting for worse time to progression after first-line chemotherapy

| Factor[a] | Univariate $\chi^2$ | Univariate P | Multivariate $\chi^2$ | Multivariate P |
|---|---|---|---|---|
| Liver metastases | 21.9 | <0.001 | 27.8 | <0.001 |
| Initial disease free interval <2 years | 14.5 | <0.001 | 18.3 | <0.001 |
| Anthracyclines *vs* other drugs[b] | 8.3 | 0.004 | 12.3 | <0.001 |

[a]The following factors were included in the regressions, but were not found to be significant ($P > 0.05$): age at diagnosis, age at start of first-line chemotherapy, stage, ductal grade, nodal status, previous endocrine therapy for advanced disease, sites of metastases at chemotherapy other than liver (Table II), local *vs* distant disease at start of chemotherapy, number of sites of distant metastases, chemotherapy regimens other than those which were anthracycline based, and previous adjuvant endocrine or chemotherapy (CMF). [b]This factor correlates with better prognosis. The remaining factors correlate with worse prognosis.

**Figure 2** Time to progression following first line chemotherapy according to presence of liver metastases.

**Figure 3** Time to progression following first line chemotherapy according to distribution of disease (locoregional *vs* metastatic).

**Table VI** Factors predicting for worse survival after first-line chemotherapy

| Factor[a] | Univariate $\chi^2$ | Univariate P | Multivariate $\chi^2$ | Multivariate P |
|---|---|---|---|---|
| Liver metastases | 41.3 | <0.0001 | 42.5 | <0.0001 |
| Initial disease free interval <2 years | 16.2 | <0.0001 | 19.8 | <0.0001 |
| Distant as opposed to local disease at CT | 25.8 | <0.0001 | 13.6 | 0.0002 |
| Miscellaneous metastatic sites[b] | 10.1 | 0.001 | 13.3 | 0.0003 |
| Increasing histological grade[c] | 20.2 | <0.0001 | 12.2 | 0.0005 |
| Increasing age at diagnosis | 4.1 | 0.04 | 5.1 | 0.02 |
| Visceral metastases (excluding liver) | 2.8 | 0.09 | 4.7 | 0.03 |
| MMC + Vinblastine *vs* other drugs | 5.0 | 0.02 | 4.0 | 0.05 |
| Anthracyclines *vs* other drugs[d] | 1.5 | 0.22 | 4.0 | 0.05 |

[a]The factors included in the regressions were as for Table V. Those not listed above were not found to be significant ($P<0.05$). [b]See Table II for a list of these sites. [c]Non-ductal histologies similar to ductal grade 2.

**Table VII** Response rates for successive chemotherapy regimens in assessable patients

| CT Regimen | CR + PR | (%) | SD | (%) | PD | (%) | ALL |
|---|---|---|---|---|---|---|---|
| First | 216 | (34) | 195 | (31) | 228 | (36) | 639 |
| Second | 30 | (16) | 61 | (33) | 95 | (51) | 186 |
| Third | 9 | (18) | 16 | (33) | 24 | (49) | 49 |
| All | 255 | (29) | 272 | (31) | 347 | (40) | 874 |

were found in this analysis. However, in 181 patients where response to both first line and second line treatment was assessed (Table VIII) response to prior chemotherapy did influence the likelihood of response to second line chemotherapy. Seventeen of 70 (24%) patients who achieved complete response (CR) or partial response (PR) with first line chemotherapy responded to second line treatment, compared with 13 of 111 (12%) who had not achieved an objective response with first line treatment ($P=0.04$).

Median time to progression (TTP) following second line chemotherapy was 2.5 months with only 15% of patients having TTP longer than 6 months and only 5% having TTP longer than 12 months (Figure 4). Median TTP for responders to second line chemotherapy (6.5 months) was longer than that for patients with SD (3.7 months; $P<0.001$). However, time to progression following second line treatment did not appear to be related to response to first line chemotherapy (Figure 5) or to first TTP. Those with first TTP longer than the median had similar second TTP to those with shorter TTP following first chemotherapy (Figure 6).

Response was assessable in 49 of 58 (84%) patients who received third line chemotherapy. Response rates and time to progression for those who received such treatment were similar to those observed for second line treatment (Table VII and Figure 4). Of the 23 assessable patients receiving third line chemotherapy having shown no previous response with 1st or 2nd line treatment five (22%) showed an objective response.

### Discussion

The primary aim of systemic treatment for patients with metastatic breast cancer is to control the disease and thereby to relieve symptoms and improve quality of life. The impact of chemotherapy on survival has never been directly assessed in this group of patients because of the difficulties inherent in conducting randomised trials with a 'no chemotherapy' arm. While it is likely that some patients survive longer as a result of receiving chemotherapy, the survival benefit attributable to chemotherapy remains uncertain. It is therefore essential that the potential benefits of systemic therapy should be carefully weighed against the likely toxicities. Achievement of objective response is associated with improvement in symptoms, and performance status (Baum *et al.*, 1980). Identification of patients who are likely to respond to chemotherapy therefore remains an important goal.

Most reports of outcome following chemotherapy for metastatic breast cancer are concerned with patients entered into clinical trials with strict eligibility criteria. In contrast to this we have examined the outcome for *all* patients who received chemotherapy at a single institution. Only 53% of the patients who have died with metastatic breast cancer had received chemotherapy for advanced disease, whereas most (74%) had received endocrine therapy. This reflects the policy adopted in this unit, which has generally been to give endocrine therapy ahead of chemotherapy, except in patients with a pattern of metastatic disease which is considered to be imminently life threatening.

The response rate (34%) to first line chemotherapy in this unselected series was, not surprisingly, lower than that frequently reported for patients entered into phase III trials, including those conducted in this unit (Rubens *et al.*, 1978; Steiner *et al.*, 1983; Amiel *et al.*, 1984; Richards *et al.*, 1992). For example, the response to anthracyclines (40%) is lower than that reported in several randomised trials (Rubens *et al.*, 1978; Steiner *et al.*, 1983; Amiel *et al.*, 1984; Perry *et al.*, 1987; Italian Multicentre Breast Study with Epirubicin, 1988; Namer *et al.*, 1990; Perez et al., 1991; Richards *et al.*, 1992), probably because patients with liver metastases and markedly abnormal liver biochemistry have been included in this study (Twelves *et al.*, 1989; Twelves *et al.*, 1991).

The higher response rates observed with anthracyclines compared to other combinations in this study may in part reflect patient selection, although the observation is consistent with the high activity and probable survival benefits seen with doxorubicin and epirubicin in this disease (A'Hern *et al.*, 1993). The other factors which were found to be related to first response, TTP and survival following first-line chemotherapy for advanced disease are similar to those

**Table VIII** Relationship between responses to first and second chemotherapy regimens

| Response to second chemotherapy | Response to first chemotherapy CR/PR | SD | PD | Total |
|---|---|---|---|---|
| CR/PR | 17 | 6 | 7 | 30 |
| SD | 21 | 20 | 18 | 59 |
| PD | 32 | 29 | 31 | 92 |
| | 70 (39%) | 55 (30%) | 56 (31%) | 181 |

**Figure 4** Time to progression according to treatment number (1, 2, 3 and 4 only).

**Figure 5** Time to progression following second line treatment according to response to first line treatment ($n = 249$).

identified in three other large studies of patients with metastatic breast cancer (Swenerton *et al.*, 1979; Namer *et al.*, 1990; Falkson *et al.*, 1991). In all four studies initial disease free interval and liver metastases (or, in the Swenerton *et al.* study, abnormal liver function tests) were identified as important prognostic factors. However, neither systemic adjuvant therapy nor number of sites of disease were identified as having prognostic importance in this study, in contrast to the other three studies. This may result from the smaller number of patients in this study, and it may also be that the specific sites of disease identified here are more important than the total number of sites. Performance status was also found to have considerable prognostic significance in two of the other three studies (Swenerton *et al.*, 1979; Namer *et al.*, 1990), but this factor was not routinely recorded in this study. With this prognostic information it is possible to identify groups of patients with high and low probabilities of response (Falkson *et al.*, 1991). However, it remains impossible to exclude a reasonable possibility of worthwhile response in individual patients. Although administration of previous adjuvant CMF was not found to be predictive for subsequent TTP after chemotherapy, the *P*-value (0.07) was of borderline significance. This result is not therefore in conflict with the results of a recent publication from this centre in which there was a small but significant ($P = 0.03$) decrease in TTP for patients who had received previous adjuvant CMF (Houston *et al.*, 1993).

Histological grade was the main factor from initial presen-

**Figure 6** Time to progression following second line treatment according to time to progression after first line treatment.

tation with breast cancer which predicted for survival after chemotherapy; this may be because this factor relates to the tumour's growth rate, and thus carries prognostic significance throughout the tumour's history.

We have confirmed that response rates and TTP following second line chemotherapy are lower than those for first line treatment. Methods for selecting patients who are likely to benefit from second line or subsequent chemotherapies have previously received little attention. Approximately one third of our patients who had received first line chemotherapy went on to receive further chemotherapy. This group included substantial numbers of patients who had experienced objective response, stable disease and progressive disease following first line therapy. As might have been expected, those who had previously responded had a higher response rate with second line therapy. However, this effect was relatively small. Perhaps surprisingly, time to progression after first line chemotherapy was not an indicator of TTP following second line therapy. Thus, although the probability of a second response can be estimated, it remains impossible to predict which individual patients are likely to derive benefit from such treatment.

In conclusion, although 30%–40% of patients will respond to chemotherapy, it is difficult to identify such patients on the basis of currently available prognostic factors. For second-line and later chemotherapies the response rate is smaller, with some 15%–20% achieving an objective response, but still no useful predictive factors to identify this group. It would seem therefore, that a clinically based judgement for each individual is still the only way of deciding whether or not to give chemotherapy for advanced breast cancer, and that it will be necessary to go on treating patients with the expectation that many of them will receive no benefit from the treatment. For the patient, deciding whether to have a particular treatment or not can be a difficult and highly subjective decision (Slevin *et al.*, 1990). These results may enable clinicians to provide patients with better information on which to make this decision in advanced breast cancer.

### References

A'HERN, R.P., SMITH, I.E. & EBBS, S.R. (1993). Chemotherapy and survival in advanced breast cancer: the inclusion of doxorubicin in Cooper type regimens. *Br. J. Cancer*, **67**, 801–805.

AMIEL, S.A., STEWART, J.F., EARL, H.M., KNIGHT, R.K. & RUBENS, R.D. (1984). Adriamycin and mitomycin C as initial chemotherapy for advanced breast cancer. *Eur. J. Cancer Clin. Oncol.*, **20**, 631–634.

BAUM, M., PRIESTMAN, T., WEST, R.R. & JONES, E.M. (1980). A comparison of subjective responses in a trial comparing endocrine with cytotoxic treatment in advanced carcinoma of the breast. In *Breast Cancer – Experimental and Clinical Methods*, Mourisden & Palshof (ed) pp. 223–228. Pergamon Press: London.

CARMO-PEREIRA, J., COST, F.A., MILES, D.W., HENRIQUES, F., RICHARDS, M.A. & RUBENS, R.D. (1991). High-dose epirubucin as primary chemotherapy in advanced breast cancer: a phase II study. *Cancer Chemother. Pharmacol.*, **27**, 394–396.

COATES, A., GEBSKI, V., BISHOP, J.F., JEAL, P.N., WOODS, R.L., SNYDER, R., TATTERSALL, M.H., BYRNE, M., HARVEY, V. & GILL, G. (1987). Improving the quality of life during chemotherapy for advanced breast cancer. A comparison of intermittent and continuous treatment strategies. *N. Engl. J. Med.*, **317**, 1490–1495.

COLEMAN, R.E., MAISEY, M.N., KNIGHT, R.K. & RUBENS, R.D. (1984). Mitoxantrone in advanced breast cancer – a phase II study with special attention to cardiotoxicity. *Eur. J. Cancer Clin. Oncol.*, **20**, 771–776.

COX, D.R. (1972). Regression models and life tables. *J. Royal Stat. Soc. (B)*, **34**, 187–220.

ENGELSMAN, E., KLIJN, J.C.N., RUBENS, R.D., WILDIERS, J., BEEX, L.V.A.M., NOOIJ, M.A., ROTMENSZ, N. & SYLVESTER, R. (1991). 'Classical' CMF versus a 3-weekly intravenous CMF schedule in post menopausal patients with advanced breast cancer. *Eur. J. Cancer Clin. Oncol.*, **27**, 966–970.

FALKSON, G., GELMAN, R., RALKSON, C.I., GLICK, J. & HARRIS, J. (1991). Factors predicting for response, time to treatment failure, and survival in women with metastatic breast cancer treated with DAVTH: a prospective eastern cooperative oncology group study. *J. Clin. Oncol.*, **9**, 2153–2161.

HAYWARD, J.L., CARBONE, P.P., HEUSON, J.C., KUMAOKA, S., SEGALOFF, A. & RUBENS, R.D. (1978). Assessment of response to therapy in advanced breast cancer. *Br. J. Cancer*, **38**, 201.

HOUSTON, S.J., RICHARDS, M.A., BENTLEY, A.E., SMITH, P. & RUBENS, R.D. (1993). The influence of adjuvant chemotherapy on outcome after relapse for patients with breast cancer. *Eur. J. Cancer*, **29A**, 1513–1518.

ITALIAN MULTICENTRE BREAST STUDY WITH EPIRUBICIN (1988). Phase III randomised study of fluorouracil, doxorubicin, and cyclophosphamide in advanced breast cancer: an Italian multicentre trial. *J. Clin. Oncol.*, **6**, 976–982.

KAPLAN, E.L. & MEIER, P. (1958). Nonparametric estimation from incomplete observations. *Am. Stat. Assoc. J.*, **53**, 457–481.

MUSS, H.B., WHITE, D.R., RICHARDS, F., COOPER, M.R., STUART, J.J., JACKSON, D.V., RHYNE, L. & SPURR, C.L. (1978). Adriamycin versus methotrexate in five-drug combination chemotherapy for advanced breast cancer: a randomised trial. *Cancer*, **42**, 2141–2148.

NAMER, M., MERCIER, M., HURTELOUP, P., BONNETERRE, J. & BASTIT, P. (1990). Prognostic factors of metastasized breast cancer patients. *Breast Cancer Res. & Treat.*, **16**, 60.

PEREZ, D.J., HARVEY, V.J., ROBINSON, B.A., ATKINSON, C.H., DADY, P.J., KIRK, A.R., EVANS, B.D. & CHAPMAN, P.J. (1991). A randomised comparison of single-agent doxorubicin and epirubicin as first-line cytotoxic therapy in advanced breast cancer. *J. Clin. Oncol.*, **9**, 2148–2152.

PERRY, M.C., KARDINAL, C.G., KORZUN, A.H., GINSBERG, S.J., RAICH, P.C., HOLLAND, J.F., ELLISON, R.R., KOPEL, S., SCHILLING, A., AISNER, J., SCHULMAN, P., WEINBERG, V., RICE, M.A. & WOOD, W. (1987). Chemohormonal therapy in advanced carcinoma of the breast: Cancer and Leukemia Group B protocol 8081. *J. Clin. Oncol.*, **5**, 1534–1545.

PETO, R., PIKE, M.C., ARMITAGE, P., BRESLOW, N.E., COX, D.R., HOWARD, S.V., MANTEL, N., MCPHERSON, K., PETO, J. & SMITH, P.G. (1977). Design and analysis of randomised clinical trials requiring prolonged observation of each patient. II. Analysis and examples. *Br. J. Cancer*, **35**, 1–39.

RADFORD, J.A., KNIGHT, R.K. & RUBENS, R.D. (1985). Mitomycin C and vinblastine in the treatment of advanced breast cancer. *Eur. J. Cancer Clin. Oncol.*, **21**, 1475–1477.

RICHARDS, M.A., HOPWOOD, P., RAMIREZ, A.J., TWELVES, C.J., FERGUSON, F., GREGORY, W.M., SWINDELL, R., SCRIVENER, W., MILLER, J., HOWELL, A. & RUBENS, R.D. (1992). Doxorubicin in advanced breast cancer: influence of schedule on response, survival and quality of life. *Eur. J. Cancer*, **28A**, 1023–1028.

RUBENS, R.D., BEGENT, R.H.J., KNIGHT, R.K., SAXTON, S.A. & HAYWARD, J.L. (1978). Combined cytotoxic and progestogen therapy for advanced breast cancer. *Cancer*, **42**, 1680–1686.

SLEVIN, M.L., STUBBS, L., PLANT, H.J., WILSON, P., GREGORY, W.M., ARMES, P.J. & DOWNER, S.M. (1990). Attitudes to chemotherapy: comparing views of patients with cancer with those of doctors, nurses, and general public. *Br. Med. J.*, **300**, 1458–1460.

STEINER, R., STEWART, J.F., CANTWELL, B.M.J., MINTON, M.J., KNIGHT, R.K. & RUBENS, R.D. (1983). Adriamycin alone or combined with vincristine in the treatment of advanced breast cancer. *Eur. J. Cancer Clin. Oncol.*, **19**, 1553–1557.

SWENERTON, K.D., LEGHA, S.S., SMITH, T., HORTOBAGYI, G., GEHAN, E.A., YAP, H., GUTTERMAN, J.U. & BLUMENSCHEIN, G.R. (1979). Prognostic factors in metastatic breast cancer treated with combination chemotherapy. *Cancer Res.*, **39**, 1552–1562.

TORMEY, D.C., WEINBERG, V.E., LEONE, L.A., GLIDEWELL, O.J., PERLOFF, M., KENNEDY, B.J., CORTES, E., SILVER, R.T., WEISS, R.B., AISNER, J. & HOLLAND, J.F. (1984). A comparison of intermittent *vs* continuous and of adriamycin *vs* methotrexate 5-drug chemotherapy for advanced breast cancer. A Cancer and Leukemia Group B study. *Am. J. Clin. Oncol.*, **7**, 231–239.

TWELVES, C.J., O'REILLY, S.M., COLEMAN, R.E., RICHARDS, M.A. & RUBENS, R.D. (1989). Weekly epirubicin for breast cancer with liver metastases and abnormal liver biochemistry. *Br. J. Cancer*, **60**, 938–941.

TWELVES, C.J., RICHARDS, M.A., SMITH, P. & RUBENS, R.D. (1991). Epirubicin in breast cancer patients with liver metastases and abnormal liver biochemistry: initial weekly treatment followed by rescheduling and intensification. *Ann. Oncol.*, **2**, 663–666.